# MiR-133a Mimic Alleviates T1DM-Induced Systolic Dysfunction in Akita: An MRI-Based Study

**DOI:** 10.3389/fphys.2018.01275

**Published:** 2018-10-10

**Authors:** Shyam Sundar Nandi, Hamid Reza Shahshahan, Quanliang Shang, Shelby Kutty, Michael Boska, Paras Kumar Mishra

**Affiliations:** ^1^Department of Cellular and Integrative Physiology, University of Nebraska Medical Center, Omaha, NE, United States; ^2^Department of Pediatric Cardiology, Children’s Hospital, Omaha, NE, United States; ^3^Department of Radiology, University of Nebraska Medical Center, Omaha, NE, United States; ^4^Department of Anesthesiology, University of Nebraska Medical Center, Omaha, NE, United States

**Keywords:** Ins2^+/-^ Akita, miR-133a, cardiac dysfunction, MRI, fibrosis, hypertrophy

## Abstract

Diabetic cardiomyopathy is a leading cause of heart failure. Developing a novel therapeutic strategy for diabetic cardiomyopathy and characterizing animal models used for diabetes mellitus (DM) are important. Insulin 2 mutant (Ins2^+/-^) Akita is a spontaneous, genetic, mouse model for T1DM, which is relevant to humans. There are contrasting reports on systolic dysfunction and pathological remodeling (hypertrophy and fibrosis) in Akita heart. Here, we used magnetic resonance imaging (MRI) approach, a gold standard reference for evaluating cardiac function, to measure ejection fraction (indicator of systolic dysfunction) in Akita. Moreover, we performed Wheat Germ Agglutinin (WGA) and hematoxylin and Eosin stainings to determine cardiac hypertrophy, and Masson’s Trichrome and picrosirius red stainings to determine cardiac fibrosis in Akita. MiR-133a, an anti-hypertrophy and anti-fibrosis miRNA, is downregulated in Akita heart. We determined if miR-133a mimic treatment could mitigate systolic dysfunction and remodeling in Akita heart. Our MRI results revealed decreased ejection fraction in Akita as compared to WT and increased ejection fraction in miR-133a mimic-treated Akita. We also found that miR-133a mimic treatment mitigates T1DM-induced cardiac hypertrophy and fibrosis in Akita. We conclude that Akita shows cardiac hypertrophy, fibrosis and systolic dysfunction and miR-133a mimic treatment to Akita could ameliorate them.

## Introduction

Diabetes mellitus (DM) has detrimental effects on multiple organs in our body (Shen et al., 2012), including the heart (Boudina and Abel, 2007). DM causes diabetic cardiomyopathy (DCM), a heart muscle disease independent of coronary artery disease, hypertension, or valvular disease, which leads to cardiac dysfunction (Rubler et al., 1972). DCM and cardiac dysfunction can be evaluated by different methods including echocardiography (Bugger et al., 2008; Basu et al., 2009; Mishra et al., 2010b; Kesherwani et al., 2015; Negishi, 2018), pressure-volume loop (Mishra et al., 2010a; Kesherwani et al., 2017) and magnetic resonance imaging (MRI) (Heijman et al., 2007; La Gerche et al., 2013). Echocardiography is a non-invasive method that utilizes ultrasound to measures mitral flow and cardiac dimension. A limitation to echocardiography is manual variability especially in positioning echocardiographic probe and/or analyses of cardiac parameters. Pressure-volume loop is widely used for assessing hemodynamic changes in the heart. In this method, a probe is inserted into the left ventricular space of the heart without touching the ventricular wall and pressure–volume relationship is recorded during contraction-relaxation cycle of the heart. A major limitation of this method is its invasive nature. MRI uses magnetic field to generate radio-frequency signals from the hydrogen atoms of the tissue that minimizes manual errors (Sun et al., 2017). MRI is a non-invasive method with least manual variability, thus considered as a gold standard reference for evaluating cardiac function (La Gerche et al., 2013).

Previous echocardiography and pressure-volume loop- based studies demonstrate cardiac dysfunction in T1DM (reduced insulin secretion) and T2DM (insulin resistance) hearts (Boudina and Abel, 2007; Jia et al., 2018; Negishi, 2018). Although T2DM is more prevalent than T1DM, the latter could have more severe impact on cardiac pathology due to high fluctuations in the blood glucose levels (Boudina and Abel, 2007; Chavali et al., 2013). Ins2^+/-^ Akita is a spontaneous, chronic, non-obese, genetic mouse model of T1DM where mutation in insulin 2 gene, which is orthologous to human insulin, causes T1DM (Yoshioka et al., 1997; Wang et al., 1999). It is relevant to humans because mutation in insulin gene causes T1DM in humans (Garin et al., 2010). Although there is no controversy on T1DM phenotype of Akita, there are contrasting reports on their cardiac pathology and dysfunction. An echocardiography and tissue Doppler-based study demonstrates an early diastolic dysfunction with preserves systolic function in 12- and 24-week Akita (Ins2 ^WT/C96Y^) (Basu et al., 2009). Another echocardiography-based study shows no significant change in cardiac parameters in 20-, 36-, and 54-week Akita, except the reduced left ventricular developed pressure in 24-week Akita that suggests a modest left ventricular dysfunction (Bugger et al., 2008). A speckle-tracking-based study reveals abnormal cardiac deformation in 12-week Akita (Zhou et al., 2018). Hemodynamic study using pressure-volume loop shows reduced ± dp/dt and ejection fraction in 12- to 14-week Akita (Kesherwani et al., 2017). Thus, there is controversy on cardiac dysfunction in Akita. Although MRI is a better method for evaluating cardiac dysfunction (La Gerche et al., 2013), to our knowledge, no MRI study has been done so far to evaluate cardiac dysfunction in Akita. Thus, the focus of our present study is to measure cardiac dysfunction in Akita using MRI. Akita develops hyperglycemia at 4-week and becomes robust hyperglycemic at 8–10 weeks (blood glucose level >500 mg/dL). Because DM at initial stage develops diastolic dysfunction and at advanced stage develops systolic dysfunction (Boudina and Abel, 2007), we sought to determine systolic dysfunction in 24-week Akita by measuring ejection fraction.

In addition to cardiac dysfunction, there is controversy on pathological cardiac remodeling in Akita. Hypertrophy and fibrosis contributes to pathological remodeling in the heart. A report on Akita heart shows no hypertrophy and fibrosis, even with elevated cardiac levels of β-myosin heavy chain, a molecular marker of hypertrophy (Basu et al., 2009). In contrast, other reports demonstrate increased cardiac hypertrophy (Chavali et al., 2014; Nandi et al., 2016) and fibrosis (Mishra et al., 2010b; Kesherwani et al., 2017) in Akita. In streptozotocin-induced T1DM, another T1DM model system, shows increased cardiac hypertrophy (Feng et al., 2010) and fibrosis (Chen et al., 2014). Other clinical and animal-based studies show pathological remodeling in diabetic hearts (Chavali et al., 2013; Jia et al., 2018; Negishi, 2018). Thus, there is a controversy on whether Akita heart shows hypertrophy and fibrosis. To resolve the controversy, we aim to measure cardiac hypertrophy and fibrosis in 24-week Akita.

Diabetes mellitus leads to heart failure (Boudina and Abel, 2007; Jia et al., 2018; Negishi, 2018). Thus, developing a novel therapeutic strategy for DCM is translationally valuable. MicroRNAs (miRNAs) are a class of tiny, non-coding, regulatory RNAs (Bartel, 2004). They are a promising therapeutic candidate for cardiovascular diseases (Mishra et al., 2009a). There are more than 800 miRNAs present in the human heart, where miR-133a is the most abundant miRNA (Leptidis et al., 2013). MiR-133a protects the heart against pathological remodeling by inhibiting hypertrophy (Care et al., 2007; Feng et al., 2010) and fibrosis (Matkovich et al., 2010; Chen et al., 2014). Notably, miR-133a is downregulated in the diabetic hearts of humans (Nandi et al., 2015) and rodents (Feng et al., 2010; Chavali et al., 2014; Nandi et al., 2016). Interestingly, overexpression of miR-133a mitigates cardiac hypertrophy (Nandi et al., 2016), fibrosis (Chen et al., 2014), and contractile dysfunction (Nandi et al., 2016) in different rodent models of diabetes. However, the role of miR-133a mimic in amelioration of cardiac remodeling and systolic dysfunction in Akita is unclear. In the present study, we aim to determine if miR-133a mimic treatment could ameliorate pathological cardiac remodeling and systolic dysfunction in Akita.

## Materials and Methods

### Animal Experiments

**FIGURE 1:**
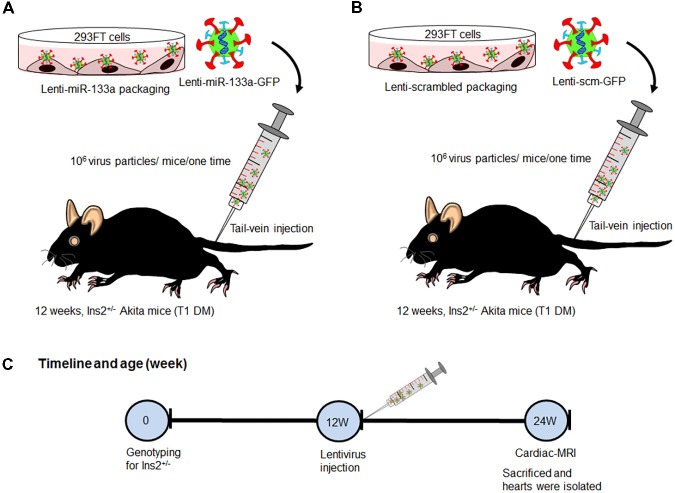
Schematic showing treatment regimen of Akita. **(A)** Lenti-miR-133a was created by packaging miR-133a mimic with lentivirus protein in 293FT cells and they were injected into Akita mice at the age of 12 weeks. **(B)** Lenti-scrambled was created in the same manner as Lenti-miR-133a except for replacing miR-133a mimic with scrambled miRNA. They were treated to Akita in the same manner as Lenti-miR-133a. **(C)** Flow schematic showing the timeline for genotyping, mice treatment, performance of cardiac-MRI, and sacrifice. WT, *N* = 5; Akita, *N* = 5; Akita+miR-133a, *N* = 5; and Akita+scm, *N* = 5.

We procured Ins2^+/-^ Akita (C57BL background) mice from The Jackson Laboratory (Bar Harbor, ME, United States) and bred them in the animal facility of the University of Nebraska Medical Center. We used 12-week male Akita and its sibling normoglycemic Ins2^+/+^ WT for experiments. Because miR-133a is reduced in Akita heart (Chavali et al., 2014), we treated Akita with miR-133a mimic. We packaged miR-133a mimic or scrambled miRNA (control) into lentivirus (Nandi et al., 2016) and administered 10^6^ virus particles/mice/one-time through tail vein injection. These mice were kept in the animal facility for 12-weeks. We performed MRI to measure cardiac dysfunction of WT, Akita, Akita treated with Lenti-miR-133a (Akita+miR) or Lenti-scrambled miRNA (Akita+scm) after 12-weeks (age of mice 24-week), and sacrificed them to collect the heart tissue for histological and molecular analyses (**Figure 1**). The breeding and experimental protocols were approved by the Institutional Animal Care and Use Committee and the Institutional Biosafety Committee of the University of Nebraska Medical Center.

### Lentivirus Packaging and Treatment

The detailed method of lentivirus packaging and delivery into mice are described in **Supplementary Material** and in our previous publication (Nandi et al., 2016). In brief, we transfected 293FT cells with GFP-tagged miR-133a or GFP-tagged scrambled miRNA vectors (cat # MmiR3445-MR03 and cat# CmiR0001-MR03, respectively, GeneCopoeia, Rockville, MD, United States) along with lentivirus protein coding genes VSVG, RSV-REV, and pMDLg/p RRE to generate miR-133a mimic-packaged (Lenti-miR-133a) or scrambled miRNA-packaged (Lenti-scrambled) lentivirus. We precipitated lentivirus by polyethylene glycol and through centrifugation, and calculated virus titer by infecting 293FT cells with different volumes of aliquoted virus.

### Cardiac Magnetic Resonance Imaging (MRI)

We anesthetized mouse with inhalation anesthesia (1.5% isoflurane in 100% O_2_), placed the mouse in a Plexiglas holder, and then placed in a whole-body-quadrature-birdcage coil for imaging. The breathing rate of the anesthetized mouse was monitored continuously using an SA Instruments (Stony Brook, NY, United States) model 1025 small animal monitoring and gating system. MRI studies were done on a 7 Tesla MRI scanner (Bruker BioSpin 70/21, Ettlingen, Germany). The MR system was run with ParaVision 5.1 including the IntraGate software for sequence acquisition and reconstruction. Studies begin with a 3-plane gradient recalled localizer (TR = 15 ms TE = 1.69 ms NA = 15, total imaging time = 30 s) to center the animal in the magnet and coil. After localization and system shimming, the following image types were obtained on each mouse. A T1 weighted coronal RARE scan (TR = 1300 ms, TE = 9 ms, RARE factor = 4, 256 × 192 acquisition matrix, 40 mm FOV, 9 slices NA = 2, total imaging time = 2.08 min) for anatomical reference and to locate the cardiac muscle. T1 weighted single slice double oblique Intragate FLASH1 (256 × 256 matrix, 30 mm FOV, TE = 2.168 ms, TR = 8.5 ms, 200 images for a total imaging time of 7.68 min). Cardiac and respiratory compensates scans were then acquired to generate images with 10 phases through the cardiac cycle (Heijman et al., 2007).

### 2D Cardiac Performance Analyses of MRI

Left ventricular (LV) function was assessed with steady state free-precession cine MR pulse sequence in the long-axis plane. CMR images were analyzed offline by a single observer (QS) using 2D cardiac performance analysis-MR (version 1.2.3.13, Edisonstrasse, Germany). The horizontal long axis was used for CMR-feature tracking analysis to derive LV longitudinal strain. The LV endocardial surfaces were manually traced by a point-and-click approach when the chamber was at its maximum size. The software, creating a region of interest, automatically generated an epicardial surface tracing. The region of interest was adjusted and automated tracking algorithm was applied. Tracking performance was reviewed to ensure accurate tracking of the ventricular myocardium, and when necessary, manual adjustments were made before the algorithm was reapplied. The software provided segmental and global values for strain in the long-axis view, as well as global ejection fraction.

### RNA Extraction and miRNA Assay

The detailed protocols for RNA extraction and miRNA assay are described in our previous publications (Mishra et al., 2009b; Chavali et al., 2014; Nandi et al., 2016). In brief, we used mirVana miRNA Isolation Kit (cat # AM1560, Life Technologies, United States) to extract miRNA. After confirming good quality RNA by NanoDrop 2000c (Thermo Fisher Scientific, Waltham, MA, United States), we made complimentary DNA using TaqMan^®^ MicroRNA reverse transcription kit (cat # 4366597, Life Technologies, United States), and then amplified miRNA by qPCR using Taqman primers specific for miR-133a (Assay ID-002246, Life Technologies, United States) and U6 SnRNA (assay ID: 001973, Life Technologies, United States). U6 snRNA was endogenous control for miR-133a assay (**Figure 5**). We used Bio-Rad CFX qPCR instrument and analyzed the results by Bio-Rad CFX Manager3.0 software (Bio-Rad Laboratories, United States).

### WGA Staining

We performed standard wheat germ agglutinin (WGA) staining on 5 μm cryosections of the heart from the four groups of mice: WT, Akita, Akita treated with miR-133a mimic (Akita+miR), and Akita treated with scrambled miRNA (Akita+scm). The details of WGA staining are elaborated in our previous publication. In brief, we prepared heart cryosections by using CryoStar NX50 (Thermo Fisher Scientific, Waltham, MA, United States) and fixed them in 4% paraformaldehyde. We washed them with phosphate buffered saline (PBS) and then incubated them with 5 μg/ml of WGA (cat # W834, Thermo Fisher Scientific, Waltham, MA, United States) for 10 min at room temperature. They were then washed, mounted with coverslip, and observed under a fluorescence microscope (EVOS, Life Technologies, United States). Representative WGA stainings of the heart from the four groups are presented in **Figures 3A, 7A**. We measured mean cardiomyocyte diameter (μm) in high magnification (40×) image. We randomly selected five areas (within the black open circle in **Figures 3C, 7C**) in the left ventricle and scored total 50 cells/per mouse from the five areas (10 cells/area). The mean cardiomyocytes diameter was quantified to determine cardiomyocytes hypertrophy.

### Hematoxylin–Eosin Staining

Cryosections of left ventricle from the above-mentioned four groups of mice were fixed in 4% paraformaldehyde. The hematoxylin and eosin (H&E) staining was performed on these fixed sections following the kit protocol (cat # HAE-1, ScyTeK Laboratories Inc., Logan, UT, United States) and observed under a bright-field microscope (Leica Microsystems, Buffalo Grove, IL, United States). The images were analyzed by the Image Pro7.0 software.

### Picrosirius Red Staining

To determine cardiac fibrosis, we performed picrosirius red staining. In brief, 10% formalin fixed left-ventricular paraffin sections (5 μm) were processed for picrosirius red staining. The reagents were Direct Red 80 (cat # 365548) and picric acid (cat # P6744-1GA) from Sigma Aldrich, St. Louis, MO, United States and glacial acetic acid (cat # A38-500) from Thermo Fisher Scientific, Waltham, MA, United States. We used standard kit protocol for this staining. We used the Tissue Core facility of the University of Nebraska Medical Center for this staining.

### Masson’s Trichrome Staining

To corroborate the picrosirius red results, we used Masson’s Trichrome staining where the blue color represents collagen (**Figures 4B, 8B**). The details of this protocol are described in our previous publication (Kesherwani et al., 2017). We followed the kit protocol (Masson’s Trichrome kit, cat # 87019, Thermo Fisher Scientific, Waltham, MA, United States) to stain paraffin sections (5 μm) of the heart. We used bright-field VWR microscope and Motic Images Plus 2 imaging tool (Motic) for imaging. We calculated perivascular (PV) and interstitial (INT) fibrosis by quantifying % blue color pixel/total pixel using Image J software, NIH. We used the Tissue Core facility of the University of Nebraska Medical Center for Masson’s Trichrome staining.

### Statistical Analyses

We evaluated cardiac dysfunction and histological analyses of the heart in random order and a blinded fashion. We presented statistical values as mean ± SD. We used two-tailed, unpaired Student’s *t*-test to determine the difference between two groups. For miR-133a assay, we used one-way ANOVA followed by Tukey’s multiple comparison test and values are presented as mean ± SEM. Graph Pad Prism 7 software was used for statistical analyses. A *P-*value of <0.05 is considered statistically significant.

## Results

### Akita Exhibits Systolic Dysfunction

**FIGURE 2:**
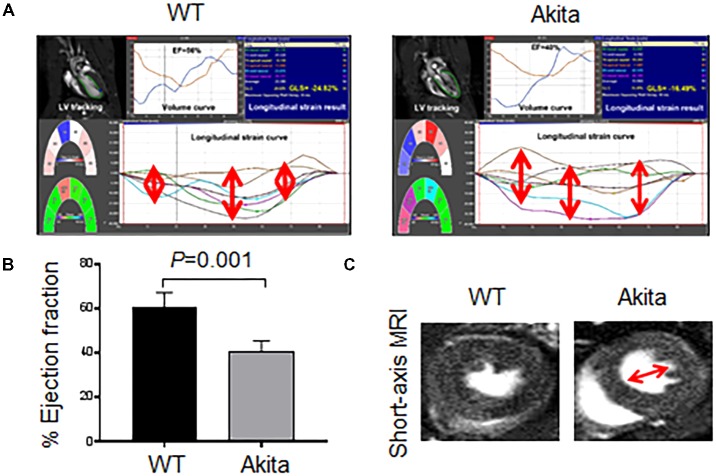
Measurement of cardiac dysfunction in Akita by MRI. **(A)** 2D cardiac performance analysis-MR tracking of left ventricle using long-axis MRI. The global longitudinal strains were recorded. **(B)** Analyses of cardiac ejection fraction by long-axis MRI. Values are represented as mean ± SD, *N* = 4–5 per group. **(C)** Representatives of short-axis images of the heart from WT and Akita mice. Two-tailed, unpaired Student’s *t*-test was used for statistical analysis. *P* < 0.05 is considered statistically significant.

To determine whether Akita exhibits systolic dysfunction, we performed MRI on WT and Akita hearts. We scored longitudinal strain in different parts of the heart (**Figure 2A**). We found that myocardial function was less aligned in Akita heart (**Figure 2A**) indicating myocardial dysfunction. Moreover, our MRI results showed reduced ejection fraction in Akita (**Figure 2B**). The short-axis view of MRI also supports cardiac dysfunction (**Figure 2C**). These findings demonstrate systolic dysfunction in Akita. Systolic dysfunction may result due to pathological cardiac remodeling, which was determined by cardiac hypertrophy and fibrosis.

### Akita Shows Cardiac Hypertrophy

**FIGURE 3:**
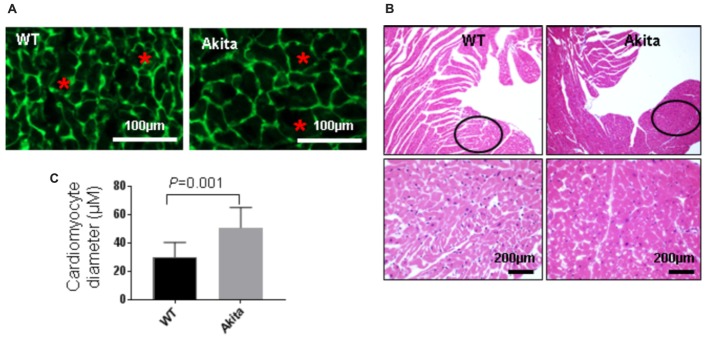
Measurement of cardiac hypertrophy in Akita. **(A)** Representative Wheat Germ Agglutinin (WGA) staining of the heart sections. Green color represents cardiomyocyte boundary and “^∗^” denotes a typical cardiomyocyte size. **(B)** Quantification of cardiomyocytes diameter. Values are presented as mean ± SD. *N* = 3 per group. Total number of cells scored for hypertrophy was 50. A minimum of five different fields of left ventricle was used from each mice. Two-tailed, unpaired Student’s *t*-test was used for statistical analysis. *P* < 0.05 is considered statistically significant. **(C)** Representative hematoxylin–eosin staining of left ventricular section. Outlined circles represent the area used for hypertrophic analysis.

To investigate if Akita has cardiac hypertrophy, we performed WGA staining. WGA stains cell membrane (green) that helps to trace cardiomyocytes boundary (**Figure 3A**). We found that cardiomyocytes size was increased in the left ventricle of Akita (**Figures 3A,B**). To support the WGA results, we performed H&E staining that also shows cardiomyocytes size. The size of cardiomyocytes was comparatively larger and space between the cardiomyocytes was less in Akita (**Figure 3C**). The left ventricle region for hypertrophy measurement was kept identical in WT and Akita (black open circles) (**Figure 3C**) and the same area was used for the WGA staining and for the quantification of cardiomyocytes area.

### Akita Shows Cardiac Fibrosis

**FIGURE 4:**
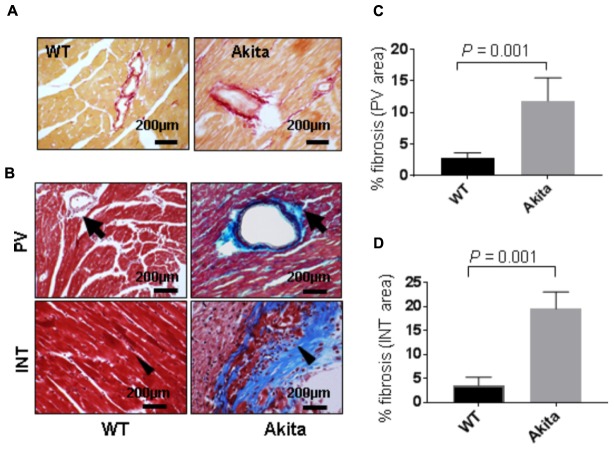
Measurement of cardiac fibrosis in Akita by picrosirius red and Masson’s Trichrome stainings. **(A)** Representative images of picrosirius red stained heart sections (left ventricle region) of WT and Akita mice. **(B)** Representative images of Masson’s Trichrome stained heart sections of WT and Akita mice. Arrow shows collagen deposition (blue) in the heart. PV, perivascular area and INT, interstitial area **(C,D)**. Quantification of percentage fibrosis (pixel blue color area/total pixel area) by Image J in the four groups. Values are presented as mean ± SD. *N* = 3 per group was randomly selected for the fibrosis analyses. Two-tailed, unpaired Student’s *t*-test was used for statistical analysis. *P* < 0.05 is considered statistically significant.

Cardiac fibrosis makes the heart stiffer and less compliant. To determine cardiac fibrosis, we performed picrosirius red staining. We found increased collagen deposition in picrosirius red stained Akita heart (**Figure 4A**), indicating increased cardiac fibrosis. To corroborate it, we performed Masson’s Trichrome staining. We observed increased collagen deposition (blue color) in Akita heart in the perivascular (PV) and the interstitial (INT) regions (**Figure 4B**). We quantified the percentage area of fibrosis in the PV (**Figure 4C**) and the INT (**Figure 4D**) regions of the heart and found that fibrosis was increased in both regions of Akita heart (**Figures 4C,D**).

Altogether, the above findings demonstrate that Akita exhibits cardiac remodeling (increased hypertrophy and fibrosis) and systolic dysfunction (reduced ejection fraction).

### MiR-133a Mimic Treatment Increases Cardiac miR-133a and Ameliorates Systolic Dysfunction in Akita

**FIGURE 5:**
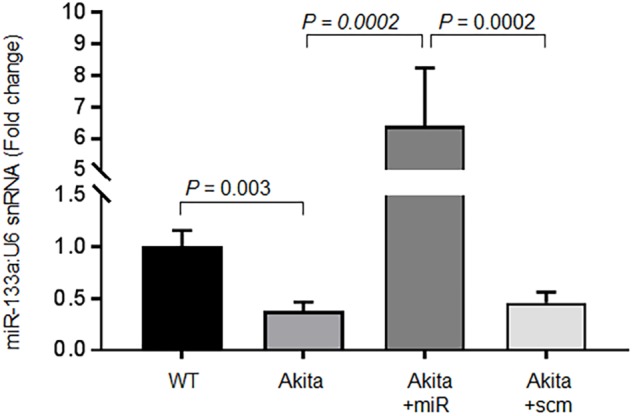
Relative miR-133a expression by the individual miR-133a assay. RT-qPCR for miR-133a was performed on the left ventricle tissue of the heart using U6 snRNA as endogenous control. Akita+miR represents Akita treated with Lenti-miR-133a, and Akita+scm represents Akita treated with Lenti-scrambled miRNA. Values are represented as mean ± SEM, *N* = 3 per group. One-way ANOVA was used for statistical analysis followed by Tukey’s multiple comparisons test. *P* < 0.05 was considered as statistically significant.

**FIGURE 6:**
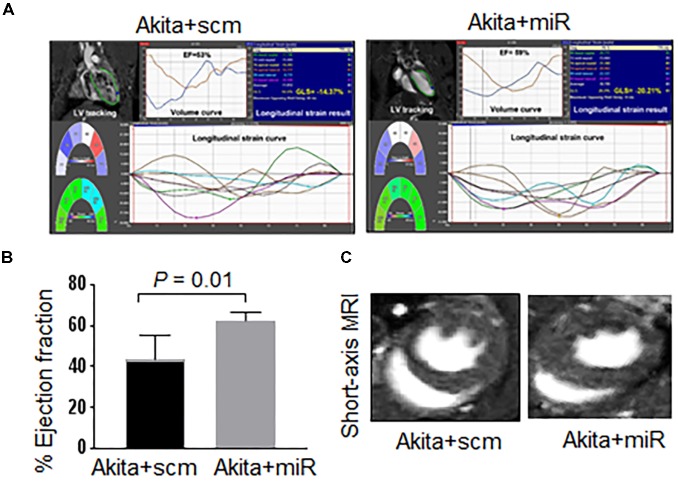
miR-133a mimic treatment improves systolic dysfunction of Akita. **(A)** 2D cardiac performance analysis-MR tracking of left ventricle using long-axis MRI. The global longitudinal strains were recorded. **(B)** Analyses of cardiac percentage ejection fraction by long-axis MRI. **(C)** Representatives of short-axis and long-axis images of the heart. Values are represented as mean ± SD, *N* = 4–5 per group. Two-tailed, unpaired Student’s *t*-test was used for statistical analysis. *P* < 0.05 is considered statistically significant.

To validate that cardiac levels of miR-133a is reduced in Akita and miR-133a mimic treatment could increase cardiac levels of miR-133a in Akita, we treated Akita with Lenti-miR-133a (**Figure 1A**) or Lenti-scrambled (**Figure 1B**). We extracted the heart and measured cardiac levels of miR-133a in the treated and the untreated groups by individual miR-133a assay (**Figure 1C**). We found that cardiac levels of miR-133a was decreased in Akita (**Figure 5**). However, Lenti-miR-133a treatment increased miR-133a level in Akita heart (**Figure 5**). These results demonstrate that miR-133a mimic treatment has potential to increase cardiac levels of miR-133a in Akita. Then, we sought to determine whether the increased miR-133a in Akita heart improves systolic dysfunction. We performed cardiac MRI on miR-133a mimic-treated (Akita+miR) and scrambled miRNA-treated (Akita+scm) Akita. We found that miR-133a mimic treatment improved the alignment of myocardial movement as reflected by the tracing (**Figure 6A**). Notably, the ejection fraction of miR-133a mimic-treated Akita was improved (**Figure 6B**), which was also supported by the short-axis view (**Figure 6C**). These findings demonstrate that miR-133a mimic treatment could mitigate systolic dysfunction of Akita.

### MiR-133a Mimic Treatment Mitigates Pathological Cardiac Remodeling in Akita

**FIGURE 7:**
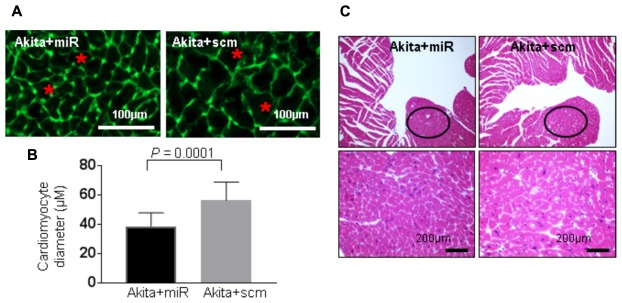
miR-133a mimic treatment mitigates cardiac hypertrophy in Akita. **(A)** Representative Wheat Germ Agglutinin (WGA) staining of the heart (left ventricle) sections. Green color represents cardiomyocyte boundary and “^∗^” denotes a typical cardiomyocyte size. **(B)** Quantification of cardiomyocytes diameter. Values are presented as mean ± SD. *N* = 3 per group. Total number of cells scored for hypertrophy was 50. A minimum of five different fields of left ventricle was used from each mice. Two-tailed, unpaired Student’s *t*-test was used for statistical analysis. *P* < 0.05 is considered statistically significant. **(C)** Representative hematoxylin–eosin staining of left ventricular section. Outlined circles represent the area used for hypertrophic analysis.

**FIGURE 8:**
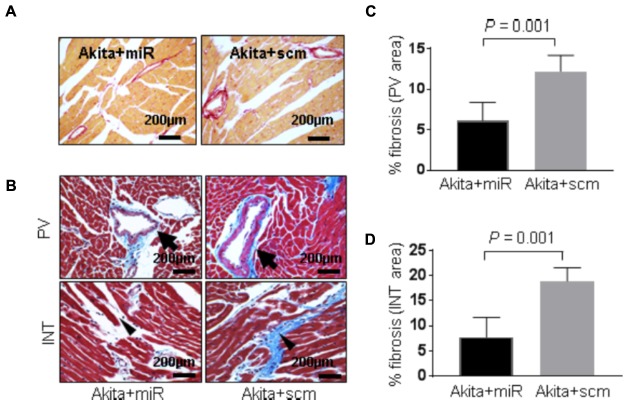
miR-133a mimic treatment alleviates cardiac fibrosis in Akita **(A)**. Representative images of picrosirius red stained heart sections (left ventricle region) of Akita treated with miR-133a mimic (Akita+miR) or scrambled miRNA (Akita+scm). **(B)** Representative images of Masson’s Trichrome staining of the heart sections of Akita+miR and Akita+scm. Arrow shows the collagen deposition (blue) in the heart. PV, perivascular area; INT, interstitial area **(C,D)**. Quantification of percentage fibrosis (pixel blue color area/total pixel area) by Image J in the four groups. Values are presented as mean ± SD. *N* = 3 per group was randomly selected for the fibrosis analyses. Two-tailed, unpaired Student’s *t*-test was used for statistical analysis. *P* < 0.05 is considered statistically significant.

DM-induced pathological remodeling contributes to cardiac dysfunction (Boudina and Abel, 2007; Chavali et al., 2013) and cardioprotective miR-133a has an anti-hypertrophy (Care et al., 2007; Feng et al., 2010) and an anti-fibrotic (Matkovich et al., 2010; Chen et al., 2014) effects. To determine if miR-133a mimic treatment mitigates cardiac remodeling in Akita, we measured cardiac hypertrophy and fibrosis in miR-133a mimic-treated (Akita+miR) and scrambled miRNA-treated (Akita+scm) mice. We measured cardiac hypertrophy by WGA staining that demarcates cell boundary (Nandi et al., 2016) and H&E staining (**Figure 7**), and cardiac fibrosis by picrosirius red and Masson’s Trichrome staining (**Figure 8**; Kesherwani et al., 2017). MiR-133a mimic treated-Akita showed decreased cardiac hypertrophy (**Figure 7**) and fibrosis (**Figure 8**) as compared to the scrambled miRNA-treated Akita (**Figures 7, 8**). These results demonstrate that miR-133a mimic treatment mitigates T1DM-induced cardiac hypertrophy in Akita.

Altogether, these results demonstrate that miR-133a mimic treatment is cardioprotective to Akita. It alleviates cardiac remodeling and ameliorates systolic dysfunction in Akita.

## Discussion

**FIGURE 9:**
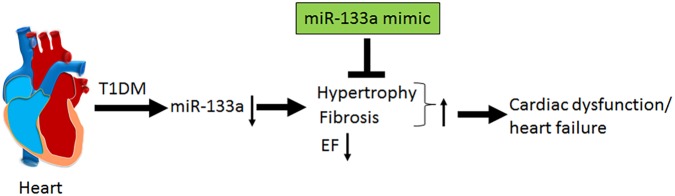
Schematics showing that T1DM decreases the cardiac levels of miR-133a, which induces cardiac hypertrophy and fibrosis and decreases percentage ejection fraction (% EF) that ultimately results in cardiac dysfunction and heart failure. MiR-133a mimic treatment mitigates T1DM-induced cardiac hypertrophy and fibrosis, increases EF and ameliorates cardiac dysfunction/heart failure.

In the present study, we demonstrate systolic dysfunction in Akita (**Figure 2**) using MRI method. MRI is a more reliable method than echocardiography and hemodynamic measurement methods (La Gerche et al., 2013). Thus, our MRI-based study gives strong basis to support cardiac dysfunction in Akita and resolves the controversy on it. To determine cardiac remodeling, we used two methods to assess cardiac hypertrophy and fibrosis. Our results demonstrate increased hypertrophy (**Figure 3**) and fibrosis (**Figure 4**) in Akita heart, supporting pathological cardiac remodeling in Akita (Mishra et al., 2010b; Chavali et al., 2014; Nandi et al., 2016; Kesherwani et al., 2017). In addition, our results demonstrate that miR-133a mimic treatment increased cardiac levels of miR-133a (**Figure 5**) and ameliorates systolic dysfunction (**Figure 6**) and pathological remodeling (**Figures 7, 8**) of Akita. These findings support the previous reports that miR-133a protects against T1DM-induced cardiac hypertrophy (Feng et al., 2010) and fibrosis (Chen et al., 2014). Based on these results, we conclude that reduced levels of miR-133a (anti-hypertrophy and anti-fibrosis) in Akita heart may contribute to cardiac hypertrophy and fibrosis resulting in cardiac dysfunction, and miR-133a mimic treatment to Akita could ameliorate T1DM-induced cardiac remodeling and systolic dysfunction (**Figure 9**).

Empirical evidences support reduced cardiac miR-133a in Akita (Chavali et al., 2014) and other models of T1DM (Feng et al., 2010; Chen et al., 2014). In our previous report, we have demonstrated that delivery of miR-133a packaged into lentivirus is able to increase the cardiac levels of miR-133a (Nandi et al., 2016). Our present results validate decreased levels of miR-133a in Akita heart (Chavali et al., 2014) and increased cardiac levels of miR-133a in miR-133a mimic-treated Akita heart (Nandi et al., 2016; **Figure 5**). Because miR-133a is largely transcribed in the heart and skeletal muscle (Liu et al., 2008), the expression of injected miR-133a mimic should be largely in these organs. Accordingly, we found ∼6-fold increase in the cardiac levels of miR-133a in Akita heart after miR-133a mimic treatment. Recently, we found that miR-133a is present in the neurons of the periventricular region of the brain (Sharma et al., 2017), thus we cannot rule out the systemic effects of miR-133a mimic treatment in Akita. This is a limitation of the present study. Future studies with cardiac-specific miR-133a transgenic mice would explain the specific impact of miR-133a overexpression in the heart on cardiac dysfunction in T1DM mice. However, the aim of this study was to determine whether the decreased miR-133a contributes to cardiac dysfunction in Akita heart. To that end, our results show decreased ejection fraction in Akita (**Figure 2**) suggesting systolic dysfunction in Akita. We believe that these findings resolve the controversy on cardiac dysfunction in Akita because MRI is a more reliable and unbiased measurement of cardiac dysfunction than the echocardiography and pressure-volume loop studies (La Gerche et al., 2013). These results also support the previous findings from echocardiography and pressure-volume loop studies that showed cardiac dysfunction in Akita (Bugger et al., 2008; Mishra et al., 2010b; Kesherwani et al., 2017). Moreover, we demonstrate that miR-133a overexpression could increase ejection fraction in Akita (**Figure 6**). Thus, miR-133a mimic could be a promising therapeutic candidate to ameliorate cardiac dysfunction in Akita and possibly in other models of T1DM.

To determine how miR-133a improves cardiac dysfunction in Akita, we aim to evaluate cardiac remodeling by measuring cardiac hypertrophy and fibrosis. Because miR-133a is an anti-hypertrophy (Care et al., 2007; Feng et al., 2010) and an anti-fibrosis (Matkovich et al., 2010; Chen et al., 2014) miRNA and downregulated in Akita heart (Feng et al., 2010; Chavali et al., 2014; Nandi et al., 2016), it is presumed that reduced cardiac levels of miR-133a may contribute to cardiac remodeling in Akita. Our results of WGA (**Figures 3A,B**) and H&E (**Figure 3C**) show increased cardiac hypertrophy in Akita. It is also documented that β-myosin heavy chain (a hypertrophy marker) (Basu et al., 2009) and matrix metalloproteinase-9 (MMP9), a fibrosis marker, (Mishra et al., 2010b) are increased in Akita heart. However, a report shows no hypertrophy and fibrosis in Akita hearts (Basu et al., 2009). One possible reason for discrepancies in the results for pathological remodeling in Akita could be strain variation (we used C57BL/6-*Ins2 ^Akita^*/J, The Jackson Laboratory stock number 003548) and/or the age (24-week) of Akita.

MiRNAs play crucial regulatory roles in biological functions (Bartel, 2004) and have emerged as a promising therapeutic candidate for cardiovascular diseases (Mishra et al., 2009a; Shah et al., 2017) and DCM (Mishra et al., 2017; Ghosh and Katare, 2018; Nandi and Mishra, 2018). Previous studies from our laboratory focusing on transcriptome analyses of Akita heart by RNA-seq and microarray (Chavali et al., 2014) and miRNA microarray (Kesherwani et al., 2017) revealed several miRNAs that are differentially regulated in Akita heart. These studies also support that miR-133a is downregulated in Akita heart. Interestingly, overexpression of miR-133a in T1DM heart improves cardiac contractility (Nandi et al., 2016) and mitigates cardiac fibrosis (Chen et al., 2014). In the present study, we demonstrate that miR-133a mimic treatment mitigates cardiac hypertrophy (**Figure 7**) and fibrosis (**Figure 8**) in Akita. Altogether, our findings provide a solid basis to demonstrate pathological remodeling in the Akita heart (**Figures 3, 4**) and also miR-133a as a therapeutic candidate to mitigate cardiac remodeling in Akita (**Figures 7, 8**).

Our results from miR-133a mimic-treated Akita mice has translational value because DM prevalence is increasing around the world (King et al., 1998; Wild et al., 2004; Arnold et al., 2016) and it increases the risk of heart failure (Haffner et al., 1998; Fox et al., 2007; Jia et al., 2018). Clinical trial shows that tight control of hyperglycemia in diabetic patients could not reduce the risk of heart failure (Castagno et al., 2011). Thus, the impetus to develop a novel therapeutic target for DCM is high. DM exacerbates mortality in patients with coronary disease (Haffner et al., 1998). Reduced cardiac miR-133a is common in humans heart failure with coronary artery disease (Barsanti et al., 2015) and with DM (Nandi et al., 2015). Thus, supplementing miR-133a could be a cardioprotective strategy to diabetic patients with coronary artery disease.

There are several critical aspects and limitations of the present study. First, miR-133a mimic treatment did not change blood glucose level in Akita (data not shown) suggesting that the mitigation of cardiac remodeling and improvement in systolic dysfunction of Akita by miR-133a mimic treatment is independent of glucose metabolic pathways. Second, the present study used a chronic model of T1DM (Akita), which may differ from acute model of T1DM (streptozotocin-induced T1DM). Thus, more studies on other models of T1DM are required to generalize the role of miR-133a in T1DM heart. Third, the study uses mice, and the results obtained from mice may not necessarily translate into humans. Therefore, further studies using human samples are required to support the role of miR-133a in DCM.

## Author Contributions

SN performed, contributed and coordinated all major experiments, analyzed the data, and wrote the manuscript. HS contributed to experiments involving lentivirus injection, mice handling, and tissue processing. MB contributed to MRI imaging. SK and QS contributed in 2D MRI data analysis. PM conceived the project, supervised the study, corrected the manuscript, and finalized the draft.

## Conflict of Interest Statement

The authors declare that the research was conducted in the absence of any commercial or financial relationships that could be construed as a potential conflict of interest.
