# Supplementary material for: MiR-133a Mimic Alleviates T1DM-Induced Systolic Dysfunction in Akita: An MRI-Based Study
Source: Front Physiol. 2018 Oct 10;9:1275. doi: 10.3389/fphys.2018.01275 (PMC6192327; doi:10.3389/fphys.2018.01275)
Supplement: Supplementary file 1 [file Table_1.docx]

**Supplemental data**

**MiR-133a Mimic Alleviates T1DM-Induced Systolic Dysfunction in Akita: An MRI-Based Study**

Shyam Sundar Nandi^1^, Hamid Reza Shahshahan^1^, Quanliang Shang^3^, Shelby Kutty^1,3^, Michael Boska^4^, Paras Kumar Mishra, ^1, 2^

^1^ Department of Cellular and Integrative Physiology, &

^2^ Department of Anesthesiology, University of Nebraska Medical Center, Omaha, NE 68198, USA.

^3^ Department of Pediatric Cardiology, Children’s Hospital, Omaha, NE 68114, USA

^4^ Department of Radiology, University of Nebraska Medical Center, Omaha, NE 68198, USA.

**Running title:** MiR-133a ameliorates Akita heart dysfunction

**Address of correspondence:**

Paras Kumar Mishra, PhD, FAHA, FCVS

Department of Cellular and Integrative Physiology

University of Nebraska Medical Center

Omaha, NE-68198-5850, USA

Tel: 1-402-559-8524, Fax: 1-402-559-4438

Email: [paraskumar.mishra@unmc.edu](mailto:paraskumar.mishra@unmc.edu)

**Lentivirus packaging and calculation of virus titer**

The 293FT cells were cultured on a 10 cm^2^ plate until it is ~90% confluent. The cells in each plate were transfected using the followings protocols: 20 μg of vector (miR-133a or scrambled) and 10 μg of each of VSVG, RSV-REV and pMDLg/p RRE (virus proteins coding genes) were mixed with 5ml of *Opti-MEM* in a 15 ml tube (designated as A) and incubated for 5 min at RT. In another 15 ml tube, 15 μL of Lipofectamine 2000 was mixed with 5 ml of *Opti-MEM* (designated as B) and incubated for 5 min at RT. Both A and B were mixed and incubated for 20 min at RT. They were slowly added to the 293FT containing plate and incubated for 24 hr. Next day, the medium was replaced with fresh 5 ml *Opti-MEM*. After 24 hr, the 5ml medium was collected and centrifuged at 3000rpm for 15 min at 4^0^C. The supernatant were collected and filtered through 0.45 μm low-protein binding disposable syringe filter (Millipore) and stored at 4^0^C. This was the first day collection. The plates were incubated with another 5ml of fresh *Opti-MEM*. Similarly, second day collection was done. Polyethylene glycol (PEG) precipitation was used for virus concentration. For preparation of PEG solution, 100 g of PEG and 6 g of NaCl was added to 250 ml of distilled water. The pH was adjusted to 7.2 and autoclaved at 15lb for 20 min. It was allowed to cool under sterilized condition. In the two days collection of virus, PEG solution was added in the ratio of 1 volume PEG to 4 volume virus collection. They were mixed and incubated at 4^0^C for 24 hr. After 24 hr, they were centrifuged at 2300 rpm for 2 hr. at 4 ^0^C. The supernatant was removed and the pellet was suspended in PBS and aliquot in PCR tubes and stored at -80^0^C until used. The 293FT cells were cultured in a 6-well plate with 2 ml medium until ~95% confluent. In different wells, different doses (2, 4, 8, 16 μL) of virus were infected and kept for 2 days at 37 ^0^C and 5% CO_2_ incubator. After two days, GFP tagged (miR-133a) cells were counted in 4 different field of view in the plate at 40X magnification. The average number of cells per 40X magnification view was counted (please see below). The titer was calculated with the formula, total number of virus particle / μL = average number of virus in 40X field of view x 4900 / the volume of virus infected in the
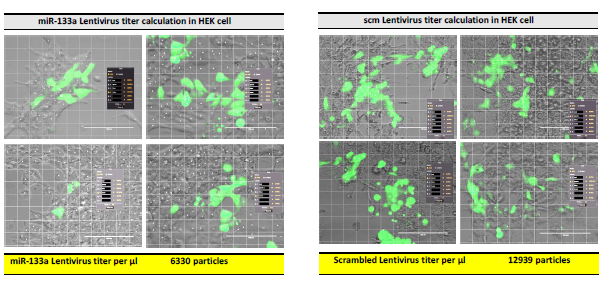
cell culture well.
